# A comparison of the efficacy and safety of complementary and alternative therapies for the primary dysmenorrhea: A network meta-analysis protocol

**DOI:** 10.1097/MD.0000000000015586

**Published:** 2019-05-13

**Authors:** Fengting Zhai, Dongmei Wang, Zhen Hua, Yuting Jiang, Dandan Wang

**Affiliations:** a The First Clinical College, Shandong University of Traditional Chinese Medicine; b The Affiliated Hospital of Shandong University of Traditional Chinese Medicine; c Qianfoshan Hospital of Shandong Province, Jinan; d Taishan Medical University, Taian, Shandong province, China.

**Keywords:** complementary and alternative therapies, primary dysmenorrhea, protocol, systematic review

## Abstract

**Background:**

There are a number of complementary and alternative therapies for the primary dysmenorrhea (PD) and their efficacy has been assessed by several systematic reviews. But only pair-wised drugs have been evaluated in the traditional meta-analyses and conflicting interpretation of results also existed among different studies. Here, a protocol for a network meta-analysis will be presented aimed to compare the efficacy and safety of different complementary and alternative therapies for PD.

**Methods:**

All randomized controlled trials of complementary and alternative therapies for the PD will be included. The primary outcomes of our interest are pain intensity and pain duration and the secondary outcomes are quality of life, clinical effective rate, and adverse events. We will search relevant database, the ongoing trial, previous relevant reviews and reference lists, and so on. The identification and selection of studies and data extraction will be conducted by two independent reviewers. We will perform a battery of pairwise meta-analyses and Bayesian network meta-analyses to assess the relative outcomes of different complementary and alternative therapies. We will use the surface under the cumulative ranking curve values and the mean ranks to get the treatment hierarchy, and then use the node-splitting method to evaluate consistency. The softwares WinBUGS 1.4.3 and STATA will be selected and the quality of the evidence will be evaluated using the Grading of Recommendations Assessment, Development and Evaluation instrument.

**Ethics and dissemination:**

This review does not require ethical approval.

**PROSPERO registration number:**

PROSPERO CRD42018107763.

## 1 Introduction

Primary dysmenorrhea (PD) is a common and often debilitating gynecological disease that usually begins in adolescence after the establishment of ovulatory cycles.^[1,2]^ It has been reported that almost 45% to 90% of women experience some menstrual pain,^[3–5]^ and 10% to 25% of reproductive women are affected by very severe PD contributing the time lost from family, work, school, and other activities.^[6,7]^ Long-term severe PD will lead to blood reflux, increase the incidence of endometriosis, and then lead to the occurrence of infertility. Besides the distress associated with dysmenorrhea, its impact on socioeconomic repercussions is significant. Several longitudinal studies on young women with dysmenorrhea have shown that the absenteeism rate ranges from 34% to 70% in these women and about 10% to 30% of all working or studying women with dysmenorrhea losing 1 to 2 working days per month, representing loss of 600 million working hours annually or up to $2 billion every year in the USA.^[8–10]^ The same is true in Sweden, although the population is only 4 million. It is reported that 50% of women have at least 1 absence from work or school due to dysmenorrhea, resulting in 230,000 lost working days. It can be seen that PD has serious global economic consequences.^[11,12]^

Now pharmacological interventions including nonsteroidal antiinflammatory drugs (NSAIDs) and hormonal contraceptives have been the mainstay of treatment for PD. NSAIDs have the main effect of immediate analgesia, however, they lack of long-term efficacy. Moreover, long-term use of NSAIDs may increase the incidence of adverse reactions, especially in digestive system and central nervous system, such as indigestion, headache, drowsiness, and poor adherence to treatment.^[13–15]^ Oral contraceptives are also commonly used in the treatment of PD, but often accompanied by weight gain, breast pain, menstrual disorders, and other adverse reactions.^[16,17]^ Besides, it is reported that frequent use of these medications have a failure rate of 20% to 25%.^[18]^ Therefore, more and more people are focusing on complementary and alternative therapies for the PD.

Complementary and alternative therapies include exercise, acupuncture, moxibustion, Chinese herbal medicines, behavioral interventions, topical heat, dietary supplements, and so on.^[19]^ Many studies and reviews have proved that the patients with PD would benefit by having complementary and alternative therapies for reducing pain treatment with minimal adverse effects. Navvabi Rigi et al^[20]^ compared the analgesic effect of heat patch containing iron chip and ibuprofen for PD through a randomized controlled trial (RCT) and found that heat patch containing iron chip had comparable analgesic effects to ibuprofen for PD. A RCT of exercise and hot water bottle in the management of dysmenorrhea conducted in India indicated that both exercise and hot water bottle could be used for girls with dysmenorrhea to provide relief from pain and menstrual distress.^[21]^ Chung et al^[22]^ conducted a systematic review of acupoint stimulation intervention for people with PD and found that acupoint stimulation, especially noninvasive acupoint stimulation, could have good short-term effects on pain in PD.^[22]^

There are so many complementary and alternative therapies for PD and their efficacy has been assessed by several systematic reviews. But the traditional pairwise meta-analyses only evaluated the direct comparison of pair-wised drugs and conflicting interpretation of results also existed among different studies. Therefore, the objective of this network meta-analysis is to compare the complementary and alternative therapies in terms of the efficacy and safety for the treatment of PD to better guide clinical practice, the management in women and health policies.

## 2 Objective

This network meta-analysis (NMA) aims to evaluate the current evidence for the efficacy and safety of complementary and alternative therapies for the PD.

## 3 Methods and analysis

Our protocol of this NMA was registered on the PROSPERO international prospective register of systematic reviews (ID=CRD42018107763). We have prepared our NMA protocol according to the Preferred Reporting Items for Systematic Review and Meta-Analysis Protocols (PRISMA-P) guidelines.^[23]^ This study will be conducted and reported in accordance with the PRISMA Extension Statement for Reporting of Systematic Reviews Incorporating Network Meta-analyses of Health Care Interventions.^[24]^

### 3.1 Eligibility criteria

This study is a systematic review with NMA of RCTs on complementary and alternative therapies in PD. All relevant RCTs using complementary and alternative therapies for PD will be included. Quasi-RCTs will be excluded such as those allocating by medical record number. The specific population, interventions, comparators, outcomes criteria are as follows.

#### 3.1.1 Population

The population included women after adolescence with PD diagnosed according to various criteria (e.g., Primary Dysmenorrhea Consensus Guidelines)^[1]^ of any duration with either moderate or severe PD, which means the pain affecting daily activity or visual analogue pain (VAS) ≥3 score. But the studies on secondary dysmenorrhea or menstrual pain caused by other factors, the participants with irregular or infrequent menstrual cycles, woman using intrauterine contraceptive device or birth control pills, and the one having a history of strenuous exercise will be excluded. We will not apply restrictions with regard to ethnic origin, region, or other characteristics.

#### 3.1.2 Interventions and comparators

RCTs of complementary and alternative therapies used in patients with PD will be included, regardless of whether the antimicrobials were tested between against placebo/control intervention or themselves (head-to-head).The complementary and alternative therapies include exercise, acupuncture, moxibustion, Chinese herbal medicines, behavioral interventions, topical heat, dietary supplements regardless of disposal method, and duration.

#### 3.1.3 Outcomes

The primary outcomes of our interest are pain intensity (the most painful day or average pain intensity on days the pain was experienced) and pain duration measured in hours. The pain intensity is measured by validated tool, such as the VAS. The VAS is a valid and reliable tool to determine the severity of pain, from 0 (no pain) to 10 (intolerable pain). The secondary outcomes are quality of life, clinical effective rate, and adverse events. Quality of life of women with PD could be measured by various questionnaires such as Short-Form Health 12, Short-Form Health 36, or any other questionnaire used to evaluate quality of life. The clinical effective rate will be calculated from the total number of patients randomized. If the studies report the clinical effective rate exceeds 2 points of time, we will compare the records obtained at the baseline with the endpoint of the treatment.

### 3.2 Search strategy

We have customized search strategies with the help of an experienced librarian for each database. We will search the following sources without restrictions for date, language, or publication status: PubMed, Cochrane Central Register of Controlled Trials (CENTRAL) Cochrane Library, and EMBASE. We will apply a combination of Medical Subject Heading (MeSH) and free-text terms incorporating database-specific controlled vocabularies and text words to implement search strategies. Table 1 shows details of the search strategy for PubMed. We will also search the ongoing trial registered in the World Health Organization's International Clinical Trials Registry Platform. Besides, the previous relevant reviews conducted on complementary and alternative therapies for PD and reference lists of included studies will also be searched.

**Table 1:**
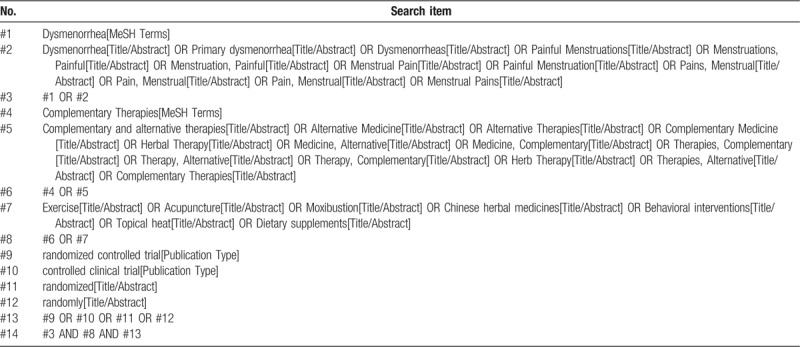
Details of the search strategy for PubMed.

### 3.3 Identification and selection of studies

All studies identified through electronic and manual searches will be imported into NoteExpress literature management software (v3.2.0.7103) and the repeated literature will be removed electronically.

The process for identification and selection of studies will be conducted in 2 separate steps:

1. Two independent authors will screen each title and abstract of the literature and exclude the apparently unrelated research.
2. Literature preliminarily included will be acquired the full text for further inspection. Also 2 independent reviewers will assess eligibility of potentially relevant articles.

Conflicts will be resolved by discussion or seeking an independent third opinion.

### 3.4 Data extraction

Two independent authors will use a pretested and standardized Microsoft Excel data extraction form to extract the data from eligible studies. In case of disagreement, an independent third reviewer will be consulted. When there is missing information that may affect eligibility, we will try to contact the original study authors. We plan to extract the following data items from each eligible study:

*Study characteristics*: title, first author, year of publication, journal, registration number to trials registries, country of conduct, number of study arms, method of randomization and blinding, country of conduct, number of centers, and funding/sponsor.

#### 3.4.1 Study participants’ characteristics

The characteristics of the study participants included gender, age, sociodemographic characteristics, sample size, number randomized to each arm, duration of disease, and disease diagnostic criteria.

#### 3.4.2 Intervention characteristics

The intervention characteristics included type of therapy, frequency, the course of treatment, and therapy setting.

#### 3.4.3 Outcome characteristics

The outcome characteristics included number of events, mean and standard errors, or standard deviation per arm.

### 3.5 Assessment of risk of bias

Risk of bias in the eligible studies will be assessed by the Cochrane Collaboration's risk of bias tool,^[25]^ which consists of 7 domains of bias relevant to the quality of RCTs. The criteria to be assessed include the following domains: random sequence generation, allocation concealment, blinding of participants, blinding of outcome assessors, incomplete outcome data, selective reporting, and other sources of bias. An assessment of risk of bias will be made for the eligible studies based on the following three levels: “low risk of bias,” “unclear risk of bias,” “high risk of bias.”

### 3.6 Data analysis

#### 3.6.1 Characteristics of the eligible studies

We will conduct descriptive statistics and describe the population characteristics of the eligible studies that include age, duration of disease, types of comparisons, duration of study, methodological variables and country, and so on.

#### 3.6.2 Analytical procedure

We will perform a battery of pairwise meta-analyses to compare the active treatments that with the same interventions and the different control arms. The Bayesian network meta-analyses will be used to assess the relevant outcomes through comparing the different complementary and alternative therapies with control conditions from both direct and indirect comparisons.

#### 3.6.3 Pairwise meta-analyses

We will perform pairwise meta-analyses for each pairwise comparison to obtain odds ratios for dichotomous outcomes or standardized mean differences for continuous outcomes, both with 95% credible intervals (CrIs). We will select a random effects model or the fixed effect model to synthesize data based on the value of I^2^. Besides, we also use the value of I^2^ to measure the amount of heterogeneity.^[26]^ If the amount of heterogeneity is characterized as high, we will explore the potential reasons for heterogeneity by subgroup analysis (see below).

#### 3.6.4 Network meta-analyses

We will perform a Bayesian network meta-analysis using the Markov chain Monte Carlo method in WinBUGS 1.4.3.^[27]^ Given that it could not produce figures, the analyses related to figures will be performed and presented using the mvmeta command in Stata 14.0.^[28]^ We will use odds ratio with 95% CrIs to report the results of dichotomous outcomes, whereas for continuous variables, we will use mean difference with 95% CrIs to assess the effect size. In consideration of the heterogeneity among the eligible studies, we will use the random effects models with vague priors for multiarm trials. It will automatically set the initial values to run the 3 Markov chains simultaneously. After that, we will use the trace plots and Brooks-Gelman-Rubin plots to ensure convergence. For 3 chains, convergence will be found to be adequate after running 1000 samples, which is discarded as “burn-in. The posterior summaries will be based on 100,000 subsequent simulations.

The ranking analysis of NMA can estimate the ranking probabilities of all treatments for each outcome. Then, we will use the surface under the cumulative ranking curve (SUCRA) values^[29]^ and the mean ranks to get the treatment hierarchy. The greater the SUCRA value, the greater the probability of becoming the best intervention. The value of SUCRA ranges from 0% to 100%, where 100% represents the greatest optimal probability and 0 represents the lowest optimal probability.

#### 3.6.5 Assessment of inconsistency

When there are 3 treatments constituting a loop, we will evaluate the inconsistency among them. For the same comparison, we will estimate the agreement between direct and indirect effect. The node-splitting method will be used to evaluate consistency in the entire network using the mvmeta command in STATA.

#### 3.6.6 Exploring heterogeneity, inconsistency, and sensitivity analyses

We expect small amounts of heterogeneity and inconsistency to be present considering the variety of study settings we plan to include. The following characteristics will be used to explore whether or not the treatment effects are robust for the 2 primary outcomes in subgroup analyses and network meta-regression:

1. Pain intensity at baseline
2. Study year
3. Study duration
4. Setting: individual vs group
5. Different types of patients, with different clinical outline concerning symptoms (if identified)

We will explore the sensitivity of our conclusions for the 2 primary outcomes, and the sensitivity will be analyzed by excluding the following aspects:

1. Studies with high risk of bias
2. Open studies
3. Studies providing published data only
4. Studies with unfair dose comparisons

#### 3.6.7 Assessment of reporting bias

We will construct a comparison-adjusted funnel plots to assess the potential publication bias and small-study effects.^[30]^ In the comparison-adjusted funnel plot, the horizontal axis will represent the difference between study-specific effect sizes and the comparison-specific summary effect. We will use visual inspection to determine the funnel asymmetry and the comparison-adjusted funnel plot should be symmetric around the zero line under the condition of the absence of publication bias and small-study effects.

#### 3.6.8 Evaluating the quality of the evidence

We will evaluate the quality of the evidence using the Grading of Recommendations Assessment, Development and Evaluation (GRADE) instrument specifically developed for network meta-analysis.^[31]^ Rating the quality of treatment effect estimates from NMA requires the best estimates from direct, indirect, and NMA (combined direct + indirect) evidences, as well as quality ratings for the direct and indirect comparisons. We will evaluate the quality of treatment effect estimates from NMA by the following 4 steps:

1. Present direct and indirect treatment estimates for each comparison of the evidence network.
2. Rate the quality of each direct and indirect effect estimate.
3. Present the NMA estimate for each comparison of the evidence network.
4. Rate the quality of each NMA effect estimate.

GRADE is characterized based on the study limitations, inconsistency, imprecision, indirectness, and publication bias. There are 4 levels classified by the GRADE for the quality of evidence: very low quality, low quality, moderate quality, and high quality.

## 4 Discussion

The evidence basis around the complementary and alternative therapies for PD has led to conflicting interpretation of results. Indeed, although several studies have assessed the comparative efficacy, the traditional pairwise meta-analyses only evaluated the direct comparison of pair-wised drugs and conflicting interpretation of results were also existed among different studies. This NMA aims to evaluate the current evidence for the efficacy and safety of complementary and alternative therapies for the PD. We will also get the treatment hierarchy of each competing treatment for the outcomes of interest. Its results will cause widespread concern: gynecologist, practice guideline developers, researchers, policymakers, as well as man whose wife or girlfriend suffered from dysmenorrhea.

Our study has several strengths. First, a comprehensive literature search will be conducted to include both published and unpublished work. Second, the GRADE will be used to rate the confidence in the estimates. Third, we will consider the potential sources of heterogeneity. Finally, a Bayesian framework will be used to pool the results and to estimate the ranking probabilities of all treatments for each outcome. On the other hand, some limitations are predictable. The publication bias exists for this review because of excluding non-English studies and some degree of clinical heterogeneity. We also hope that our findings will provide evidence and fill the gaps to reduce the uncertainty about the ranking of the interventions in terms of effectiveness and safety and encourage further research to enhance clinical decision-making.

## Author contributions

**Conceptualization:** Fengting Zhai, Dongmei Wang.

**Data curation:** Fengting Zhai, Zhen Hua, Yuting Jiang.

**Formal analysis:** Fengting Zhai, Zhen Hua.

**Funding acquisition:** Dongmei Wang.

**Methodology:** Fengting Zhai, Zhen Hua, Yuting Jiang, Dandan Wang.

**Resources:** Dandan Wang.

**Software:** Fengting Zhai, Zhen Hua, Dandan Wang.

**Supervision:** Yuting Jiang.

**Writing – original draft:** Fengting Zhai.

**Writing – review & editing:** Fengting Zhai, Dongmei Wang.

## References

[1] Burnett M Lemyre M No. 345-primary dysmenorrhea consensus guideline. J Obstet Gynaecol Can 2017;39:585–95. 28625286 10.1016/j.jogc.2016.12.023

[2] Rosenwaks Z Seegar-Jones G Menstrual pain: its origin and pathogenesis. J Reprod Med 1980;25:207–12. 7001019

[3] Rencz F Péntek M Stalmeier PFM Bleeding out the quality-adjusted life years: evaluating the burden of primary dysmenorrhea using time trade-off and willingness-to-pay methods. Pain 2017;158:2259–67. 28767507 10.1097/j.pain.0000000000001028

[4] Proctor M Farquhar C Diagnosis and management of dysmenorrhoea. BMJ 2006;332:1134–8. 16690671 10.1136/bmj.332.7550.1134 PMC1459624

[5] Unsal A Ayranci U Tozun M Prevalence of dysmenorrhea and its effect on quality of life among a group of female university students. Ups J Med Sci 2010;115:138–45. 20074018 10.3109/03009730903457218 PMC2853792

[6] Banikarim C Chacko MR Kelder SH Prevalence and impact of dysmenorrhea on Hispanic female adolescents. Arch Pediatr Adolesc Med 2000;154:1226–9. 11115307 10.1001/archpedi.154.12.1226

[7] László KD Gyrffy Z Ádám S Work-related stress factors and menstrual pain: a nationwide representative survey. J Psychosom Obstet Gynaecol 2008;29:133–8. 18484442 10.1080/01674820701804423

[8] Burnett MA Antao V Black A Prevalence of primary dysmenorrhea in Canada. J Obstet Gynaecol Can 2005;27:765–70. 16287008 10.1016/s1701-2163(16)30728-9

[9] Sundell G Milsom I Andersch B Factors influencing the prevalence and severity of dysmenorrhoea in young women. Br J Obstet Gynaecol 1990;97:588–94. 2390501 10.1111/j.1471-0528.1990.tb02545.x

[10] Dawood MY Nonsteroidal anti-inflammatory drugs and changing attitudes toward dysmenorrhea. Am J Med 1988;84:23–9. 10.1016/0002-9343(88)90473-1 3287908

[11] Hofmeyr GJ Bassin J Dysmenorrhoea. Topics in Obstetrics and Gynaecology. Johannesburg: Julmar Communications; 1996 269–74.

[12] Jones AE Managing the pain of primary and secondary dysmenorrhoea. Nurs Times 2004;100:40–3. 15045780

[13] Marjoribanks J Ayeleke RO Farquhar C Nonsteroidal anti-inflammatory drugs for dysmenorrhoea. Cochrane Database Syst Rev 2015;(7):CD001751. 10.1002/14651858.CD001751.pub3 PMC6953236 26224322

[14] Tziona P Theodosis-Nobelos P Rekka EA Medicinal chemistry approaches of controlling gastrointestinal side effects of non-steroidal anti-inflammatory drugs. Endogenous protective mechanisms and drug design. Med Chem 2017;13:408–20. 28185540 10.2174/1573406413666170209123433

[15] Abaraogu UO Igwe SE Tabansi-Ochiogu CS Effectiveness of SP6 (Sanyinjiao) acupressure for relief of primary dysmenorrhea symptoms: a systematic review with meta and sensitivity analyses. Complement Ther Clin Pract 2016;25:92–105. 27863617 10.1016/j.ctcp.2016.09.003

[16] Shi S Klotz U Clinical use and pharmacological properties of selective COX-2 inhibitors. Eur J Clin Pharmacol 2008;64:233–52. 17999057 10.1007/s00228-007-0400-7

[17] Uysal G Akkaya H Cagli F A comparison of two different oral contraceptives in patients with severe primary dysmenorrhoea. J Obstet Gynaecol 2018;38:828–32. 29537325 10.1080/01443615.2017.1410533

[18] Iwasaki Y Morita A Iwasawa T A nonpungent component of steamed ginger-[10]-shogaol-increases adrenaline secretion via the activation of TRPV1. Nutr Neurosci 2006;9:169–78. 17176640 10.1080/110284150600955164

[19] Campbell MA McGrath PJ Non-pharmacologic strategies used by adolescents for the management of menstrual discomfort. Clin J Pain 1999;15:313–20. 10617260 10.1097/00002508-199912000-00008

[20] Navvabi Rigi S Kermansaravi F Navidian A Comparing the analgesic effect of heat patch containing iron chip and ibuprofen for primary dysmenorrhea: a randomized controlled trial. BMC Womens Health 2012;12:25. 22913409 10.1186/1472-6874-12-25 PMC3492023

[21] Chaudhuri A Singh A Dhaliwal L A randomised controlled trial of exercise and hot water bottle in the management of dysmenorrhoea in school girls of Chandigarh, India. Indian J Physiol Pharmacol 2013;57:114–22. 24617160

[22] Chung YC Chen HH Yeh ML Acupoint stimulation intervention for people with primary dysmenorrhea: systematic review and meta-analysis of randomized trials. Complement Ther Med 2012;20:353–63. 22863651 10.1016/j.ctim.2012.02.008

[23] Shamseer L Moher D Clarke M Preferred reporting items for systematic review and meta-analysis protocols (PRISMA-P) 2015: elaboration and explanation. BMJ 2015;350:g7647. 25555855 10.1136/bmj.g7647

[24] Hutton B Salanti G Caldwell DM The PRISMA extension statement for reporting of systematic reviews incorporating network meta-analyses of health care interventions: checklist and explanations. Ann Intern Med 2015;162:777–84. 26030634 10.7326/M14-2385

[25] Higgins JPT Green S Cochrane Handbook for Systematic Reviews of Interventions Version 5.1.0 [updated March 2011]. The Cochrane Collaboration 2011;http://handbook.cochrane.org/

[26] Higgins JPT Altman DG Deeks JJ Higgins JPT Chapter 9: Analysing data and undertaking meta-analyses. John Wiley & Sons, Cochrane Handbook for Systematic Reviews of Interventions.. Chichester (UK): 2011.

[27] Lunn DJ Thomas A Best N WinBUGS – a Bayesian modeling frame work: concepts, structure, and extensibility. Statist Comput 2000;10:325–37.

[28] White IR Barrett JK Jackson D Consistency and inconsistency in network meta-analysis: model estimation using multivariate metaregression. Res Synth Methods 2012;3:111–25. 26062085 10.1002/jrsm.1045 PMC4433771

[29] Salanti G Ades AE Ioannidis JP Graphical methods and numerical summaries for presenting results from multiple-treatment meta-analysis: an overview and tutorial. J Clin Epidemiol 2011;64:163–71. 20688472 10.1016/j.jclinepi.2010.03.016

[30] Egger M Smith GD Schneider M Bias in meta-analysis detected by a simple, graphical test. BMJ 1997;315:629–34. 9310563 10.1136/bmj.315.7109.629 PMC2127453

[31] Guyatt G Oxman AD Akl EA GRADE guidelines: 1. Introduction-GRADE evidence profiles and summary of findings tables. J Clin Epidemiol 2011;64:383–94. 21195583 10.1016/j.jclinepi.2010.04.026

